# The respiratory manifestations in patients with primary Sjögren’s syndrome: is interstitial lung disease related to disease activity?

**DOI:** 10.55730/1300-0144.5513

**Published:** 2022-04-19

**Authors:** Özlem ÖZDEMİR IŞIK, Ayten YAZICI, Ayşe ÇEFLE

**Affiliations:** Division of Rheumatology, Department of Internal Medicine, Faculty of Medicine, Kocaeli University, Kocaeli, Turkey

**Keywords:** Sjögren’s syndrome, lung, interstitial lung disease

## Abstract

**Background/aim:**

Sjögren’s syndrome is a systemic, autoimmune disease and can affect many organs and systems. In this study, we aimed to evaluate the respiratory manifestations, and the association between interstitial lung disease (ILD) and disease activity in primary SS (pSS) patients.

**Materials and methods:**

The study design was retrospective cross-sectional, and 151 patients followed up with a diagnosis of pSS between 2004 and 2019 were included in the study. Demographic and clinical data, laboratory results, chest radiographs and thorax computed tomography (CT) results were obtained from patient files and hospital imaging system. Thorax CT was requested from all patients with respiratory complaints and abnormalities in physical examination and pulmonary function test. Disease activity was calculated with EULAR pSS disease activity index (ESSDAI) and clinical European League Against Rheumatism SS Disease Activity Index (clin-ESSDAI).

**Results:**

In our study, 97% of pSS patients were female, and the mean age was 55.9 ± 12 years, disease onset age was 45.5 ± 12.1 years, disease duration was 10.7 (1–38) years. According to CT findings of 120 patients, 35% had nodules, and 13.3% had ILD (62.5% nonspecific interstitial pneumonia, 25% lymphocytic interstitial pneumonia, 12.5% usual interstitial pneumonia). Bronchiectasis, emphysema, sequelae fibrotic changes, and pleural thickening was found in 3.3%, 5.8%, 15.8%, and 1.7% of patients, respectively.

It was observed that there was a significant relationship between the presence of ILD and persistent cough, mediastinal LAP, low DLCO, high ESSDAI and clin-ESSDAI scores reflecting disease activity.

**Conclusion:**

The most common pulmonary manifestation in our patients was ILD. ILD was observed more frequently in patients with moderate and severe disease activity. Some of the ILD patients were diagnosed while they were asymptomatic. Even if they are asymptomatic, it is important to follow up the patients with physical examination, spirometry, DLCO and thorax CT.

## 1. Introduction

Primary Sjögren’s syndrome (pSS) is a systemic, autoimmune disease characterized by lymphoplasmacytic infiltration of exocrine glands [1]. Pulmonary involvement affects 9%–12% of patients; however, with bronchoalveolar lavage, pulmonary function tests and computed tomography (CT) data, it is seen that 75% of the patients are affected in the last period [2]. Although the most common pulmonary involvement is airway abnormalities in pSS, interstitial pneumonias are also common [3]. Pulmonary hypertension and pleural effusion are also among the conditions that can be seen, but it is less frequently seen than other connective tissue diseases [4–6]. Pulmonary involvement is a condition that significantly affects the quality of life. In this study, we aimed to present the respiratory manifestations of pSS patients we followed up in our outpatient clinic. In addition, clinical and laboratory parameters, including respiratory manifestation, were compared according to disease activity.

## 2. Materials and methods

The study design was retrospective cross-sectional, between January 2020 and June 2020. Patients who were followed up with the diagnosis of pSS in our outpatient clinic between 2004 and 2019 were included in the study. The data of 151 patients who were diagnosed with pSS according to the classification criteria of the European-American consensus group [7]. Clinical, demographic, laboratory findings, the drugs and pulmonary function tests [spirometry and diffusing capacity of the lungs for carbon monoxide (DLCO)] were examined from their files. Chest radiographs and thorax CT results were accessed via hospital imaging system. The study was retrospective, and chest X-ray of all patients was evaluated by a rheumatologist during their outpatient follow-up, and tomography was not requested from patients who did not have respiratory complaints and whose physical examination and pulmonary function tests (forced vital capacity (FVC) > 80, forced expiratory volume in one second (FEV1) > 80, DLCO > 80) were normal. Thus, thorax CT results of 120 patients were obtained. All thorax CTs were evaluated by a radiologist specialized in lung imaging. Disease activity was calculated with EULAR pSS disease activity index (ESSDAI) [8] and clinical European League Against Rheumatism Sjögren’s Syndrome Disease Activity Index (clin-ESSDAI) [9]. In some patients, because the diagnosis of interstitial lung disease (ILD) was made before the diagnosis of pSS, disease activities were evaluated during the study period, not at the time of ILD diagnosis. The study was performed according to the Declaration of Helsinki.

### 2.1 Statistical analysis

Statistical evaluation was performed with IBM SPSS 20.0 (IBM Corp., Armonk, NY, USA) package program. Compliance with normal distribution was evaluated with Kolmogorov–Smirnov and Shapiro–Wilk tests. Numerical variables were given as mean ± standard deviation and median (min-max), and categorical variables as percentages. Differences between groups were determined by independent sample t-test and ANOVA for numerical variables with normal distribution, and Mann–Whitney U and Kruskal–Wallis tests for numerical variables that do not have normal distribution. Binary logistic regression analysis was used to determine the factors affecting the variable of interest. Relationships between categorical variables were evaluated using chi-square analysis. In the test of two-sided hypotheses, p <0.05 was considered statistically significant.

## 3. Results

The mean age of 151 pSS patients (97% of the patients were women) was 55.9 ± 12 years, the mean disease onset age was 45.5 ± 12.1 years, the mean disease duration was 10.7 (1–38) years, and the follow-up period was 45.8 ± 33.8 months. Fever was present in 5.3% of the patients, weight loss in 7.9%, and night sweats in 1%. A total of 88.1% of the patients had dry mouth, 86.1% had dry eyes, 16.6% had parotitis, 28.5% had arthritis, 19.9% had Raynaud phenomenon, 29.1% had lymphadenopathy (LAP), 20.5% had smoking, 22.5% had hypergammaglobulinemia (hyperIgG), 9.3% had malignancy and 72% had decreased uptake and excretion in salivary gland scintigraphy. Salivary gland biopsy confirmed the diagnosis in 66.1% of patients. The Schirmer test was positive in 82.2% of patients, rheumatoid factor (RF), antinuclear antibody (ANA), anti SS-A and anti SS-B antibodies were 65.2%, 90.7%, 62.3%, 50.3%, respectively. Smoking rate among the patients was 20.5%. The effect of smoking on clinical data and thorax CT results were evaluated. A significant relationship was found between smoking and the development of emphysema (p = 0.001). The rate of persistent cough was significantly higher in smokers as expected (p = 0.013). Since 31 patients did not have thorax CT results, all analyzes except the general information given above were performed on 120 patients and presented. When the thorax CT results of 120 patients were reviewed, no finding was observed in 27.5% of the patients. At least one symptom was present in 72.5% of the patients.

According to the CT findings, 35% had nodules in the lung. The size of the nodules was less than 1 cm and the patients were followed for the nodule size progression. Hilar and subcarinal lymph nodes were present in 12.5% of the patients, and their sizes ranged from 5 mm to 15 mm. Bronchoscopy was performed for mediastinal LAP in 2 patients, and the results of bronchoscopic biopsy were evaluated as reactive changes. Two patients had lung cancer (CA), and they died. Pulmonary manifestations according to thorax CT and spirometry results of pSS patients are shown in Table 1.

Cough and shortness of breath were observed in 13.8% of the patients with respiratory manifestation. ILD was detected in 75% of symptomatic patients. According to the results of thorax CT, ILD was detected in 13.3% of the patients. Lung biopsy is generally not recommended in pSS patients with ILD, as HRCT and histopathological findings are well correlated [10]. Therefore, the diagnosis of ILD was made according to CT results. The follow-up period of ILD patients was 4.16 ± 3.5 years. The patients were diagnosed with ILD 4 ± 3.1 years after the diagnosis of pSS. A total of 31.3% of the patients were diagnosed with ILD before the diagnosis of pSS.

Smoking history was in 12.5% of ILD patients and persistent cough in 43.8%. All patients with ILD received steroid (0.5–1 mg/kg/daily) therapy for a short time. Two patients received cyclophosphamide (6 cycles/monthly) therapy for active alveolitis, and azathioprine (AZA) was used as maintenance therapy for 7 years. While 9 patients were treated with AZA (1.5–2 mg/daily), and one treated with mycophenolate mofetil (MMF) (2 g/daily) for eight years. One patient received Rituximab (RTX) treatment (two 1000 mg doses, 15 days apart/every 6 months) for three years due to an increase in interstitial involvement under AZA treatment.

When we evaluated the ESSDAI activity scores, it was seen that 65.8% of our patients had low disease activity, 25.8% had moderate disease activity, and 8.3% had high disease activity. When the patients were evaluated according to disease activity levels, a significant statistical difference was observed in patients with ILD, respiratory manifestations and, mediastinal LAP. In particular, 70% of patients with high disease activity had ILD, and this rate was 25.8% in patients with moderate disease activity and 1.3% in patients with low disease activity. Respiratory manifestations were observed to be higher in patients with moderate and high disease activity. However, it should be kept in mind that not all respiratory manifestations may be associated with pSS (such as lung cancer, tuberculous sequel). It was observed the disease onset age of the patients with low disease activity was lower than the patients with moderate and high disease activity. There was no difference in the duration of the disease and the mean age of the patients in all three activity groups. Comparison of clinical, demographic and laboratory findings according to disease activity is presented in Table 2.

Patients with pSS with and without ILD were compared according to their clinical and laboratory findings. All patients with ILD were women. The mean ages and disease duration were similar in both groups. Persistent cough was significantly higher in patients with a diagnosis of ILD. Mediastinal LAP was higher in patients with ILD than in patients without ILD. Malignancy development was found to be higher but not statistically significant in patients with a diagnosis of ILD.

ESSDAI and clin-ESSDAI scores were evaluated in both groups in terms of the disease activity. ESSDAI and clin-ESSDAI scores of patients with interstitial lung disease were significantly higher than the group without ILD (p = 0.000) (Figure). When the respiratory function tests of both groups were compared, it was seen that the DLCO test results were significantly lower in patients with ILD (p = 0.028), and the difference between FVC and forced expiratory volume in 1 s (FEV1)/FVC results were not statistically significant. Both groups did not differ from each other in terms of mean age and duration of disease. Two patients with pulmonary arterial hypertension (PAH) were in the non-ILD group. The data of patients with and without ILD are compared in Table 3.

In the logistic regression analysis performed to determine the risk factors affecting the presence of ILD, it was observed that there was a significant relationship with persistent cough, mediastinal LAP, low DLCO, high ESSDAI and clin-ESSDAI scores reflecting disease activity. In the multivariate analysis, a significant relationship was found only between high clin-ESSDAI scores and the presence of ILD (Table 4).

Laboratory and clinical findings of 16 patients with ILD were presented in Table 5, but statistical analysis could not be performed due to the small number of patients in the subgroups. FVC and DLCO values were found to be better in lymphocytic interstitial pneumonia (LIP) subgroup than other subgroups in ILD. The ESSDAI and clin-ESSDAI score were higher LIP and usual interstitial pneumonia (UIP) patients compared to nonspecific interstitial pneumonia (NSIP) patients (Table 5).

## 4. Discussion

In our study, the respiratory complaints and the respiratory manifestations detected by imaging methods of pSS patients followed up in our outpatient clinic, and the relationship between these findings (especially ILD) with disease activity score were presented. The respiratory manifestation that we observe in Sjögren’s syndrome are varied, and not all of these findings are related to disease and disease activity. However, ILD is associated with disease activity. In the literature, the incidence of respiratory manifestations is estimated to be 10% 1 year after the diagnosis of Sjögren’s syndrome, and the rate rises to 20% after 5 years [11]. Our ILD patients were diagnosed 4 ± 3.1 years after the diagnosis of pSS is consistent with the literature. A total of 31.2% of ILD patients were diagnosed when they were asymptomatic, so it is important to evaluate patients with examination, spirometry, DLCO and imaging methods, even if they are asymptomatic. The respiratory manifestations were evaluated separately, regardless of their relationship with disease activity.

In 7.1% of the pSS patients, shortness of breath was the reason for admission to the outpatient clinic. The rate of persistent cough among patients was 19.2%. The persistent cough rate has been reported as 41%–61% in patients with Sjögren’s syndrome [12], but this rate was found to be significantly lower in our study. However, persistent cough was significantly higher in the ILD group. The reasons might include airway involvement, bronchial inflammation, abnormal mucociliary clearance, gastroesophageal reflux [13].

Nodules were detected in 23% of the patients in thorax CT. It is known that bronchial mucosa-associated lymphoid tissue (BALT) lymphoma [14] and pulmonary amyloidosis [15] may have nodular appearance in the lung parenchyma. Therefore, patients are followed up in terms of increase in nodule size and nature.

Mediastinal LAP may be related to follicular bronchiolitis, interstitial lung disease, and pulmonary lymphoma [13]. Mediastinal lymphadenopathies were observed more in patients with interstitial lung disease and high disease activity score in our study. The high disease activity in most of the ILD patients also overlaps with this situation. Bronchoscopy was performed in 2 cases due to increased LAP size and the biopsy results were reported as benign changes.

Most of our patients had low disease activity and it was observed that patients with low disease activity were younger. It was thought that the patients who were diagnosed and treated at an earlier age, and who were regularly followed-up in the outpatient clinic have lower disease activities in the long term, although they had similar disease durations due to better disease control.

Malignancy development was found to be higher in patients with a diagnosis of ILD. ESSDAI and clin-ESSDAI scores of patients with ILD were significantly higher than the group without ILD (p = 0.000). Due to the relationship of ILD with disease activity and the heavy burden of disease, the rate of malignancy was considered to be high. According to a meta-analysis, hospital-based studies showed higher RRs for malignancy than population-based studies. It is thought that hospitalized patients with pSS have more severe disease which suggests that more severe pSS is higher probability of malignancy due to disease burden [16].

Interstitial lung disease was detected in 13.3 % of our patients, and the vast majority of them were in the NSIP pattern that supports the literature [17]. Although there are studies showing that pSS patients with ILD are older than patients without ILD [18–20], in our study, there was no significant difference between both groups in terms of the mean age (p = 0.167), and disease duration (p = 0.824). Some studies also reported that older age at disease onset and longer disease duration were associated with a higher risk of pulmonary involvement in pSS [21,22]. In our study, the ILD group had significantly older disease onset, but no significant relationship was found in the logistic regression analysis. In ILD subgroups, it was observed that UIP patients were older, had older disease onset and longer disease duration. Also, the patients with UIP had higher disease activity. ESSDAI and clin-ESSDAI scores were higher in the UIP and LIP groups than in the NSIP group. There was no significant difference between ILD subgroups in terms of pulmonary domain of ESSDAI. In addition, when we look at the pulmonary domain of ESSDAI scores of patients with ILD, it was observed that 87% had moderate activity and 13% had high activity.

The course of the disease in the NSIP pattern may be uncertain [23]. Due to the diagnosis of NSIP, 40% of the patients received AZA, 10% CyC, 10% MMF and short-term steroid treatment. Steroids are generally recommended for NSIP patients [13]. The mean follow-up period was 2.9 ± 2.83 years. A total of 50% of the NSIP patients were diagnosed with ILD before the diagnosis of pSS. Studies showing that the use of AZA, CyC and RTX in the treatment of NSIP support our data [13].

LIP is the most characteristic of pSS and is marked by lymphoplasmacytic infiltration tissue (such as interstitium, alveolar space) and lymphoid aggregates. A polyclonal or monoclonal gammopathy may be observed in ILD patients and LIP can be a precursor to a BALT lymphoma [24]. Patients are followed more carefully in this respect. In our study, LIP patients accounted for 25% of all ILD cases. LIP patients consisted of younger patients and earlier onset of disease compared to other ILD subgroups. The mean follow-up period was 6.7 ± 4.5 years. Although LIP patients have moderate disease activity in terms of the ESSDAI pulmonary domain, ESSDAI score have been found to be high due to the lymphoid load they carry, with the effect of the extra pulmonary domains. When these patients were diagnosed with LIP, they were asymptomatic in terms of respiratory findings. All LIP patients received steroid and they used AZA for extra pulmonary involvement.

Restrictive ventilation defect is seen in pulmonary function tests of pSS-ILD patients [25]. Spirometry might be done early after disease onset, even in asymptomatic patients. The reduction in DLCO appears to be the most common abnormality in ILD [13]. In the early phase of ILD, a preserved FVC is observed with a decrease in DLCO. DLCO is more sensitive in predicting the presence of ILD, FVC may be more useful when assessing the extent of the disease [26]. Although FVC was lower in the ILD group than non-ILD in our study, no statistical significance was observed. However, DLCO was significantly lower in the ILD group (p = 0.028). DLCO was lower in ILD subgroups, especially in NSIP and UIP groups.

Studies have shown that there is an association between ILD and ANA, anti SS-A, anti SS-B antibodies positivity positivity, hyperIgG, RF positivity and lymphopenia [27, 19, 20]. In our study, there was no significant relationship between ILD and ANA, anti SS-A, anti SS-B antibodies, and RF positivity (not evaluated as titer). In addition, there was no significant difference between the patients with and without ILD in terms of hyperIgG.

The number of patients with airway abnormalities (3%) and chronic obstructive pulmonary disease (COPD) (1%) in our study was quite low. Although the airway abnormality as the most common finding of pulmonary involvement in pSS patients [12], the most common involvement in our study was ILD. Airway abnormality is associated with destruction or cellular infiltration of exocrine glands in pSS, and trachea and bronchi are also affected [12]. It can be difficult to understand whether the airway abnormality is due to in pSS or is due to some other reason, such as smoking. Although it has been shown in some studies conducted with pSS patients that the incidence of COPD increased 1.4 times compared to the general population, such a result was not obtained in our study [28].

PAH is an important and severe complication, which is encountered in many collagen tissues disorders. There are case reports [29, 30] on the presence of PAH in pSS and one study [31] using echocardiography. In our study, 2 patients were diagnosed with PAH by right heart catheterization. In addition, in 2 patients, pulmonary systolic pressure measured assessment only with echocardiography, and it was found 35 mmHg. For that reason, the latest two patients were not included in the statistical analyses in term of PAH. All of these four patients had no ILD.

The limitations of our study were its retrospective design, small sample size, and short follow-up time. Due to the small number of ILD patients, statistical analysis could not be performed on ILD subgroups. The strength of the study was that the physical examination, spirometry, DLCO and thorax CT results of 120 pSS patients were performed in a single center and by experts in the field. In addition, the scarcity of studies evaluating the relationship between disease activity and ILD makes this study important. Although disease activity was low in most of our patients, the activity level was found to be high in ILD patients. More studies are needed on the relationship between disease activity and organ involvement.

## 5. Conclusion

Respiratory manifestations are seen more frequently in pSS with the effect of innovations in imaging methods. Pulmonary involvement is a condition that significantly affects the quality of life. Especially, ILD is an important cause of morbidity and mortality in pSS. Early diagnosis is important. The fact that most of the patients with radiological findings are asymptomatic and even some of the patients diagnosed with ILD are asymptomatic have shown us the importance of physical examination, spirometry, DLCO, chest X-ray and, if necessary, tomography. Patients should be informed about long-term regular follow-up and treatment compliance.

## Figures and Tables

**Figure:**
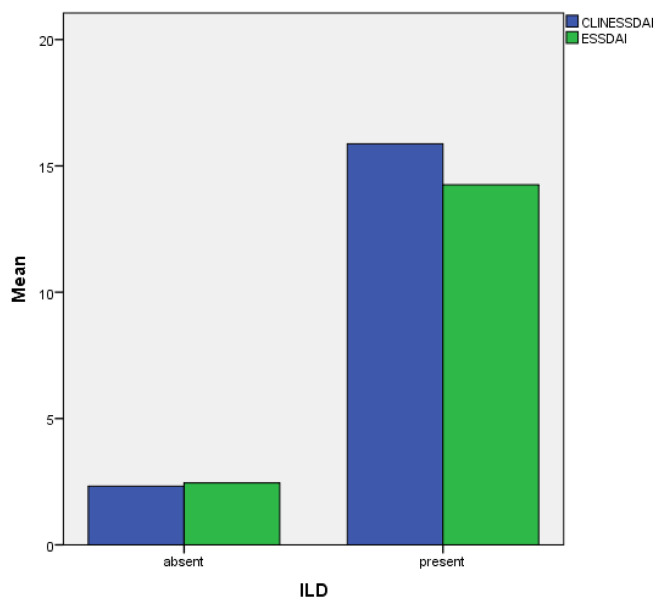
Comparison of disease activity in groups with and without ILD.

**Table 1:** Respiratory manifestations according to thorax CT and spirometry results of pSS patients.

n: 120	n (%)
Nodule	42 (35)

Mediastinal LAP	15 (12.5)

Interstitial Lung Disease	16 (13.3)
NSIP	10 (62.5)
LIP	4 (25)
UIP	2 (12.5)

Bronchiectasis	4 (3.3)

Segmental atelectasis	7 (5.8)

Emphysema	7 (5.8)

Sequela fibrotic change	19 (15.8)

Tuberculous sequel	2 (1.7)

Airway disease	4 (3.4)

Lung cancer	2 (1.7)

Pleural effusion	2 (1.7)

COPD	2(1.7)

pSS: primary Sjögren’s syndrome, LAP: lymphadenopathy, NSIP: nonspecific interstitial pneumonia, LIP: lymphocytic interstitial pneumonia, UIP: usual interstitial pneumonia, COPD: chronic obstructive pulmonary disease.

**Table 2:** Comparison of data according to disease activity.

n (%)	Low (n: 79)	Moderate (n: 31)	High (n: 10)	p
Age (mean±std)	55.7±11.85	61.77±11.04	59±14.4	*0.054* ≈
Disease onset age	44.91±12.18	51.43±11.76	48.92±12.84	*0.038* ≈
Disease duration	129.38±67.12	124.16±65.37	121±50.60	0.886≈
Smoking	17(25)	11(35.5)	0	NA
Dry mouth	66(83.2)	28(90.3)	10(100)	0.278
Dry eyes	69(86.1)	28(90.3)	9(90)	0.807
Vasculitis	3(3.8)	2(6.5)	1(10)	0.636
Neuropathy	0	4(12.9)	4(40)	NA
Myositis	0	1(3.2)	1(10)	NA
Parotitis	14(17.7)	5(16.1)	2(20)	0.958
Raynaud phenomenon	13(16.5)	9(29)	1(10)	0.239
Respiratory manifestations	49(62)	29(93.5)	9(90)	*0.002*
Mediastinal LAP	7(8.9)	4 (12.9)	4(40)	*0.003*
ILD	1(1.3)	8 (25.8)	7(70)	*0.000*
LAP	24(30.4)	7(22.6)	6(60)	0.083
Malignancy	7(8.9)	6(19.4)	1(10)	0.300
ANA (+)	72 (91.1)	28(90.3)	9(90)	0.987
Anti SS-A antibody	53(67.1)	16(51.6)	6(60)	0.316
Anti SS-B antibody	42(53.2)	12(38.7)	6(60)	0.317
HyperIgG	20(25.3)	6(19.4)	5(50)	0.154
Hypocomplementemia	10(15.6)	3(10.7)	0	NA

≈ Anova, LAP: lymphadenopathy, ILD: interstitial lung disease, ANA: antinuclear antibody, hyperIgG: hypergammaglobulinemia, NA: not available.

**Table 3:** Clinical and laboratory data of pSS patients with and without ILD.

n (%)	pSS with ILD n: 16	pSS without ILD n: 104	p
Age (mean ± std)	60.4 ± 11.3	55.7 ± 12.3	0.167^T^
Disease duration (mean ± std)	10.3 ± 6	10.7 ± 5.4	0.824^T^
Disease onset age (mean ± sd)	50.2 ± 9.9	45.3 ± 12.2	*0.147* ^T^
DLCO	60.1 ± 20.4	75 ± 15.1	*0.028* ^T^
FVC	87.9 ± 22.8	92.9 ± 19.2	0.232^T^
FEV1/FVC	103.3 ± 16.2	105.2 ± 12.3	0.495^T^
ESSDAI *	13 (5–28)	1 (0–19)	*0.000* ≈
Clin-ESSDAI *	14.5 (5–31)	0 (0–22)	*0.000* ≈
Smoking	2 (12.5)	26 (28)	0.233
Cough	7 (43.8)	16 (15.4)	*0.014*
Dry mouth	14 (87.5)	90 (86.5)	1
Dry eyes	13 (81.3)	92 (88.5)	0.421
Vasculitis	1 (6.3)	5 (4.8)	0.585
Raynaud	4 (25)	19 (18.3)	0.506
Parotitis	2 (12.5)	19 (18.3)	0.735
Mediastinal LAP	6 (37.5)	9 (8.7)	*0.005*
Malignancy	4 (25)	10 (9.6)	0.092
ANA (+)	15 (93.8)	94 (90.4)	1
Anti SS-A antibody	10 (62.5)	65 (62.5)	1
Anti SS-B antibody	10 (63)	50 (48.1)	0.421
HyperIgG	5 (31.3)	26 (25)	0.556
PAH	-	2 (1.7)	NA

≈ Mann-Whitney U test used,

T Student’s t-test,

* Since the data are not normally distributed, median (min-max) values are given. DLCO: diffusing capacity of the lungs for carbon monoxide, FVC: forced vital capacity, FEV1/FVC: forced expiratory volume in 1 s/forced vital capacity, ANA: antinuclear antibody, ILD: interstitial lung disease, LAP: lymphadenopathy, ESSDAI: EULAR primary Sjögren’s syndrome disease activity ındex, clin-ESSDAI: clinical European League Against Rheumatism Sjögren’s Syndrome Disease Activity Index, hyperIgG: hypergammaglobulinemia, PAH: pulmonary arterial hypertension, NA: not available.

**Table 4:** Factors affecting ILD according to the logistic regression model.

	*Univariate*		*Multivariate*	
	OR (%95CI)	p	OR (%95CI)	p
Cough	4.28 (1.39–13.14)	*0.011*		
Mediastinal LAP	6.33 (1.87–21.48)	*0.003*		
DLCO	0.96 (0.919–0.998)	*0.041*		
ESSDAI	1.42 (1.23–1.63)	*0.000*		
Clin-ESSDAI	1.41 (1.22–1.63)	*0.000*	1.51 (1.19–1.91)	*0.001*

LAP: lymphadenopathy, DLCO: diffusing capacity of the lungs for carbon monoxide ESSDAI: EULAR primary Sjögren’s syndrome disease activity ındex, clin-ESSDAI: clinical European League Against Rheumatism Sjögren’s Syndrome Disease Activity Index.

**Table 5:** Clinical and laboratory findings of ILD subgroups patients.

n (%)	NSIP (n = 10)	LIP (n = 4)	UIP (n = 2)
Age	61.5 **±** 11.8	58 ± 12.9	68 ± 2.7
Disease duration	9.2 **±** 7.2	11.8 **±** 3	12.9 **±** 1.3
Disease onset age	52.3 **±** 10.1	46.2 **±** 10.3	55.1 **±** 14
Dry mouth	9 (90)	3 (75)	2 (100)
Dry eyes	8 (80)	3 (75)	2 (100)
Raynaud	3 (30)	1(25)	-
Parotitis	1 (10)	-	1 (50)
Arthritis	1 (10)	3 (75)	1 (50)
ANA positivity	9 (90)	4 (100)	2 (100)
Anti SS-A antibody positivity	5 (50)	4 (100)	1 (50)
Anti SS-B antibody positivity	5 (50)	4 (100)	1 (50)
Cough	4 (40)	1 (25)	2 (100)
Smoking	2 (20)	-	-
Malignancy	3 (30)	-	1 (50)
HyperIgG	2 (20)	2 (50)	1 (50)
ESSDAI	10 (5–15)	21 (13–28)	18(15–21)
Clin-ESSDAI	12(5–18)	22 (15–31)	19.5 (17–22)
FVC	85(61–146)	94 (88–107)	65(60–70)
DLCO	58(40–84)	76(69–84)	58(50–65)

NSIP: nonspecific interstitial pneumonia, LIP: lymphocytic interstitial pneumonia, UIP: usual interstitial pneumonia, HT: hypertension; ANA: antinuclear antibody, ILD: interstitial lung disease; hyperIgG: hypergammaglobulinemia, ESSDAI: EULAR primary Sjögren’s syndrome disease activity index, FVC: forced vital capacity, DLCO: diffusing capacity of the lungs for carbon monoxide, clin-ESSDAI: clinical European League Against Rheumatism Sjögren’s Syndrome Disease Activity Index.
